# Conversion of superior bread wheat genotype HD3209 carrying *Lr19/Sr25* into CMS line for development of rust-resistant wheat hybrids

**DOI:** 10.1038/s41598-024-65109-x

**Published:** 2024-06-19

**Authors:** Abhimanyu Singh Malik, Nand Kishore Sharma, Ajay Kumar Chandra, Parvesh Kumar, Sandhya Tyagi, K. Raghunandan, Niranjana Murukan, Niharika Mallick, Shailendra Kumar Jha, Vinod

**Affiliations:** Division of Genetics, ICAR-Indian Agricultural Research Institute, New Delhi, 110012 India

**Keywords:** Wheat, Hybrid breeding, Line conversion, Cytoplasmic male sterility, Rust resistance, Plant breeding, Plant genetics

## Abstract

Hybrid development is one of the most promising strategies for boosting crop yields. Parental lines used to create hybrids must have good per se performance and disease resistance for developing superior hybrids. Indian wheat line HD3209 was developed by introducing the rust resistance genes *Lr19/Sr25* into the background of popular wheat variety HD2932. The wheat line HD3209 carrying *Lr19/Sr25* has been successfully and rapidly converted to the CMS line A-HD3209, with 96.01% background genome recovery, based on selection for agro-morphological traits, rust resistance, pollen sterility, and foreground and background analyses utilizing SSR markers. The converted CMS line A-HD3209 was completely sterile and nearly identical to the recurrent parent HD3209. Based on high per se performance and rust resistance, the study concludes that the derived CMS line A-HD3209 is promising and can be employed successfully in hybrid development.

## Introduction

In wheat (*Triticum aestivum* L.), leaf rust is considered the most prevalent disease caused by the pathogenic fungus *Puccinia triticina* Eriks (*Pt*). During severe outbreaks, this biotrophic fungus results in substantial yield losses ranging from 30 to 50% while affecting susceptible cultivars^1,2^. Due to its greater prevalence and frequent occurrence in wheat-growing regions worldwide, leaf rust is considered the most devastating of the three wheat rusts^3^. Exploiting host plant resistance by developing resistant cultivars has become essential in managing leaf rust disease without effective chemical and biological control agents^4^. Eighty-three leaf rust resistance genes have been uncovered and catalogued in the wheat gene symbol database^5^. About half are native to wheat, while others are introgressed from wild and related species^6^. Identifying novel resistance genes against multiple pathogen races is one of the most efficient techniques to breed for durable leaf rust resistance^2^. The most efficient method for introducing a specific gene(s) to superior wheat cultivars or parental lines is through marker-assisted backcross breeding (MABB). In wheat, the success of MABB for rust resistance has been well-proven ^1,6–8^. In an earlier program from our lab, the wheat line HD3209^9^ carrying leaf rust resistance gene *Lr19* having linkage with *Sr25* has exhibited a high level of disease resistance. In addition, the wheat has achieved high yield potential through continuous breeding efforts in developing varieties via the pedigree breeding method and utilizing the available rust resistance genes. The further enhancement in yield can be supplemented by developing hybrid cultivars. The hybrids have enhanced the yield potential of several cross-pollinated crops like maize, rye, and sorghum^10^ over self-pollinated crops like wheat, rice, oats, and barley^11^. Rice is the only self-pollinated crop among cereals where successful hybrid breeding has enhanced the yield^12–14^. In India, the hybrid development using the cytoplasmic male sterility (CMS) approach in wheat was initiated by Indian council of agricultural research (ICAR), but no hybrid could have been developed. Although some wheat hybrids like Pratham 7070 and Pratham 7272 were released for commercial cultivation in central and peninsular India in 2002 (Mahyco), they were later discontinued. Further, with superior varieties having better yield and per se performance than hybrids, the popularity of hybrids could not be achieved^15,16^. In recent years, with encouraging results from several countries like the USA, China, Mexico, and the European Union, hybrid breeding in India has been reinitiated in wheat crops by ICAR, New Delhi.

The hybrid seed needs deliberate crossing of the female (seed) parent with the male (pollen) parent in isolation. To get a pure hybrid seed, the pollen from the female parent is taken out via detasseling in the case of maize, emasculation in the case of vegetables and high-value seed, and by the application of chemical hybridizing agents (CHA) ^17,18^. However, using CHA in wheat hybrid breeding has limited results and has many operational difficulties in commercial seed production ^19^.

The CMS system offers cost-effective breeding efforts for hybrid seed production. Further, CMS lines in hybrid wheat breeding have gained momentum with the development of the male sterility system using sources like *T. timopheevii*^20^. The three-line hybrid system involves a CMS/A-line as the seed/female parent, restorer/R lines used as the pollen/male parent and a maintainer/B-line to maintain the A-line^21^. The economic viability of hybrid can be achieved only with superior parents having high per se performance and having good combining ability to give better heterosis over the superior parent and, more desirably, over the best commercial check^22^. The high per se performance ensures the profitability of seed production, leading to the availability of hybrid seeds even to marginal farmers. Developing superior parents for hybrid wheat can contribute to disease resistance and other desirable agronomic traits^23,24^. Besides, a dominant resistance gene, even in one parent, can ensure hybrid resistance. In addition, it can also complement the resistance contributions from other parents. Hence, it reduces the requirements to have resistance to all prevalent diseases in a single line, as in the case of a pure line variety^25^. Thus, considering the yield performance of HD3209 and the effectiveness of the *Lr19/Sr25* combinations, the present study aimed to rapidly convert superior wheat line HD3209 into a CMS line carrying *Lr19/Sr25*. The conversion of superior lines with rust resistance can provide an alternative option for hybrid breeding to have parental lines with better per se performance and source of rust resistance in hybrid development.

## Results

### Development of cytoplasmic male sterile line A-HD3209

**Figure 1:**
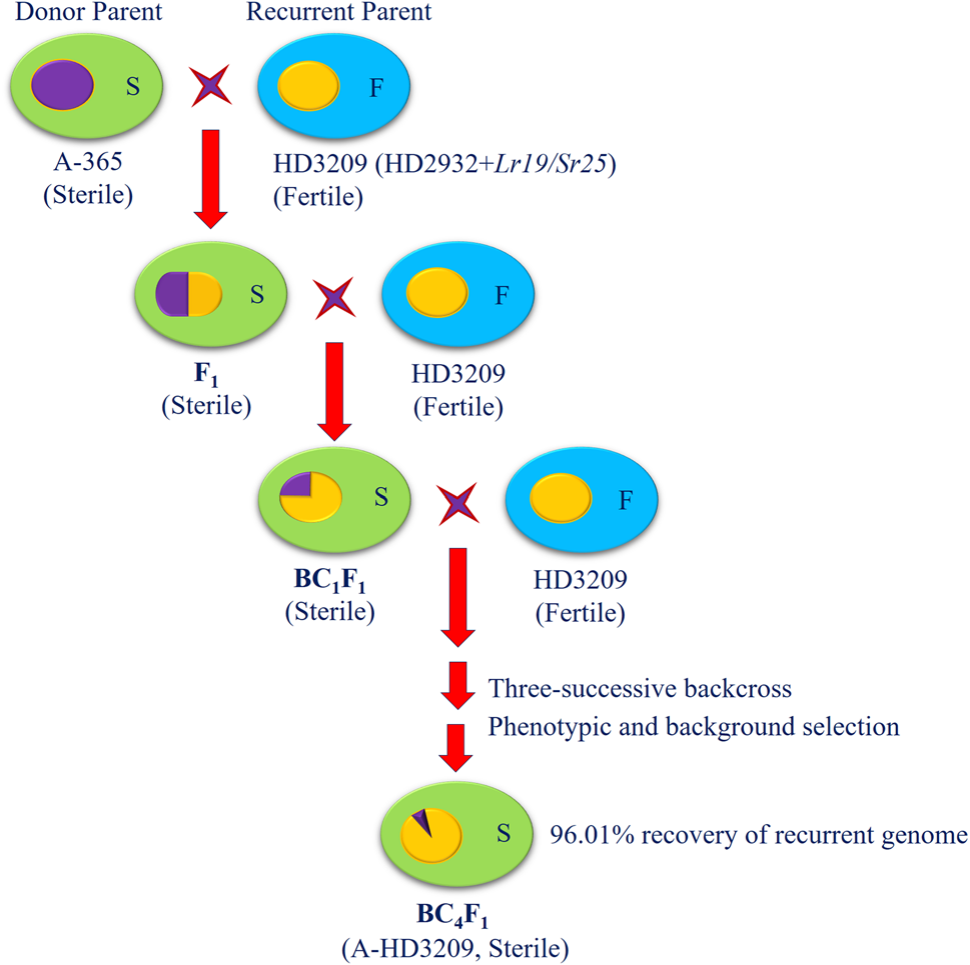
Systematic backcross breeding scheme for development of CMS line A-HD3209.

Plenty of F_1_ seeds were obtained from the cross A-365 × HD3209, five of which were planted. The line HD3209 was used as a male parent and crossed with CMS line A-365 as a female parent. The A-365 is a CMS line carrying *timopheevii* cytoplasm and was found to have complete sterility and perfect maintenance. The F_1_ between A-365 and HD3209 was completely sterile and resistant to leaf rust. Further, backcross breeding was initiated to transfer cytoplasmic male sterility into rust-resistant recurrent parent HD3209. Only the best plants were selected from the backcross progenies for further evaluation and generation advancement, while the rest were rejected based on their agro-morphological performance (Table 1). Five of 100 BC_1_F_1_ plants were phenotypically selected based on visual agro-morphological traits similar to recurrent parent and backcrossed with recurrent parent HD3209 thrice to raise BC_2_F_1_, BC_3_F_1_ and BC_4_F_1_ generations. Following the cycles of backcrossing and selection, 25 BC_4_F_1_ plants from the selected BC_3_F_1_ plant were further cross-checked for pollen sterility through cytogenetic profiling. Finally, all BC_4_F_1_ plants with completely sterile pollen have been designated as a CMS line A-HD3209. The details of the backcross breeding scheme employed for developing the CMS line A-HD3209 are presented in Fig. 1.

**Table 1:** The details of backcrossed plants grown and selected for generation advancement.

S. no	Crossing/backcross generation	Number of plants grown	Number of plants selected
1	F_1_	5	5^a^
2	BC_1_F_1_	100	5^a^
3	BC_2_F_1_	100	5^a^
4	BC_3_F_1_	100	1
5	BC_4_F_1_	25	25

^a^Number indicates the plants bulked to grow 100 plants at given generations.

### Cytogenetic study for pollen sterility

**Figure 2:**
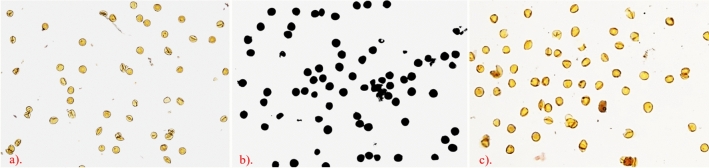
Cytogenetic analysis for pollen viability of (**a**) A-365, (**b**) B-HD3209, and (**c**) derived CMS line A-HD3209 at the anthesis stage.

Cytogenetic analysis for pollen fertility at the anthesis stage of BC_4_F_1_ plants has revealed complete sterility (Fig. 2). The mature pollen grains that were completely spherical and intensely pigmented were considered fertile. At the same time, unstained or stained but withered were classified as sterile. The cytoplasmic donor parent A-365 appears completely sterile, while B-HD3209 is consistently pigmented with complete fertility. The derived CMS line A-HD3209 showed complete sterility in BC_4_F_1_ generation.

### Phenotyping of leaf rust resistance

The backcross progenies till BC_3_F_1_ were screened under field conditions against the mixed pathotypes and showed resistance with infection type (IT) ranging from 0 to 5R. The leaf rust-resistant parent HD3209 was highly resistant. It displayed the fleck reaction (;) at 5% leaf area (5R), and susceptible parent A-365 produced a susceptible phenotypic response with ITs 40S against mixture of leaf rust pathotypes (Table 2). All the backcross progenies were resistant in all the generations as the recurrent parent was resistant, and the backcross progenies will be either homozygous or heterozygous resistant. The selected homozygous BC_4_F_1_ plants were resistant, with no visible leaf rust infection in the field. The developed A-HD3209 with ITs 5R showed no visible leaf rust infection, indicating the effectiveness of the dominant alien *Lr19/Sr25* genes from the recurrent parent.

**Table 2:** Infection type (ITs) of leaf rust incidence level recorded in the parental lines and selected homozygous BC_4_F_1_ plants.

S. no	Leaf rust resistance	Pathotypes	Level of infection		
			A-365	B-HD3209	A-HD3209
1	Adult plant resistance	Mixed	40S	5R	5R
2	Seedling	77-5	33+	0;	0;
3		77-9	33+	0;	0;
4		104-2	33+	0;	0;
5		12-5	33+	0;	0;

Furthermore, the recurrent parent HD3209 line showed seedling resistance for leaf rust against all four pathotypes of *P. triticina* used in the study with IT “0;" while the A-365 line was susceptible with IT "33+". The A-HD3209 has also displayed a similar disease reaction with an IT of 0; under glasshouse conditions at the seedling stage against all four pathotypes tested separately (Fig. 3).

**Figure 3:**
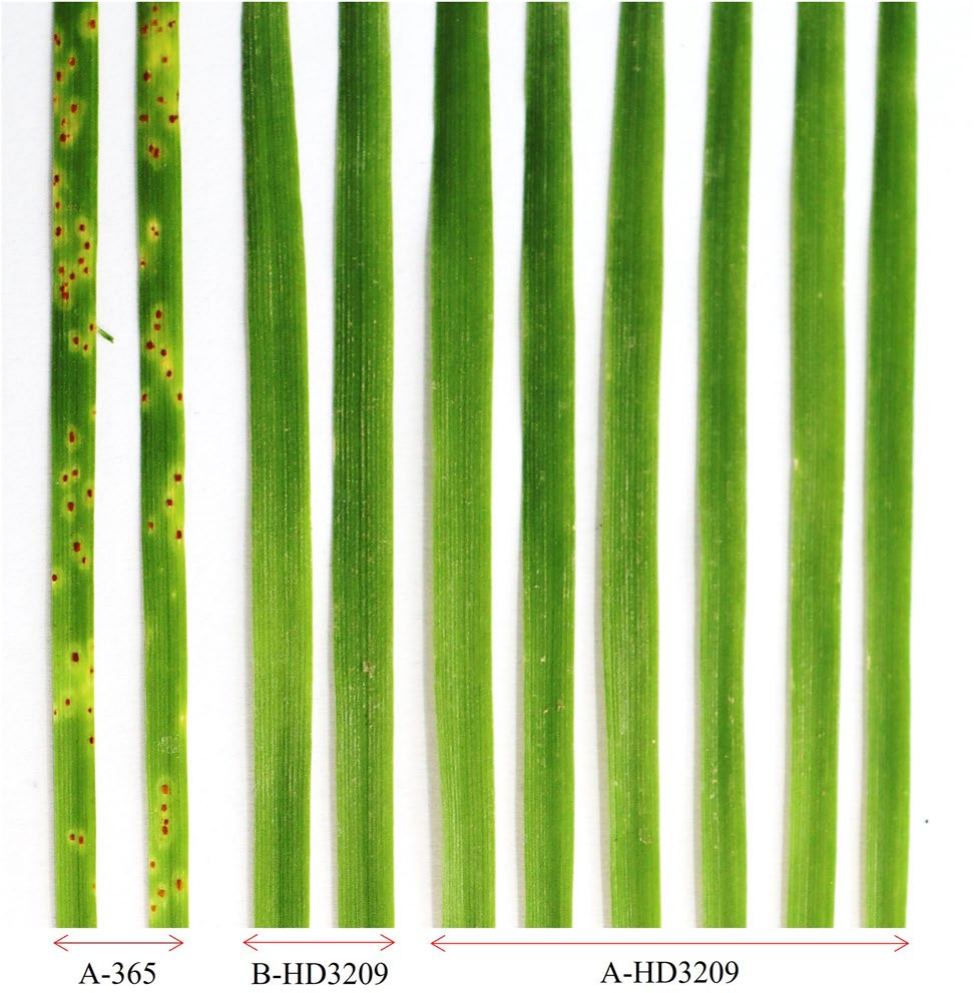
Seeding reaction for leaf rust response using prevalent Indian pathotype 77-5 of *Puccinia triticina* in A-365, B-HD3209, and derived CMS line A-HD3209 under glasshouse condition.

### Foreground and background analysis

**Figure 4:**
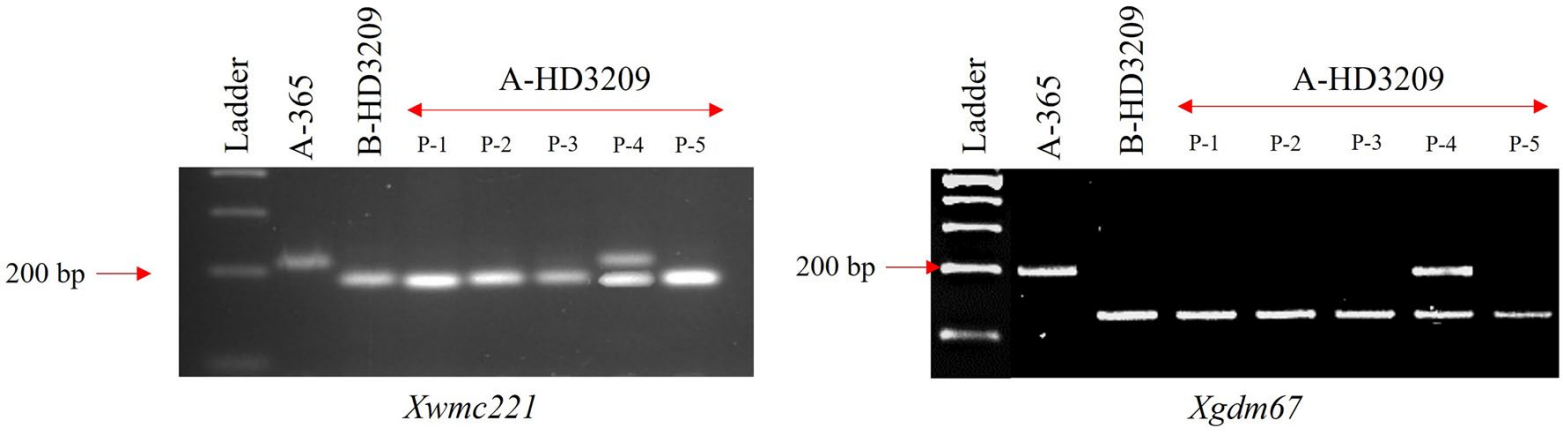
Amplification profile of *Lr19/Sr25*-linked codominant foreground markers, *Xwmc221*, and *Xgdm67* [from left to right: ladder (100 bp); P_1_, A-365; P_2_, B-HD3209, and five plants of derived CMS line A-HD3209].

**Figure 5:**
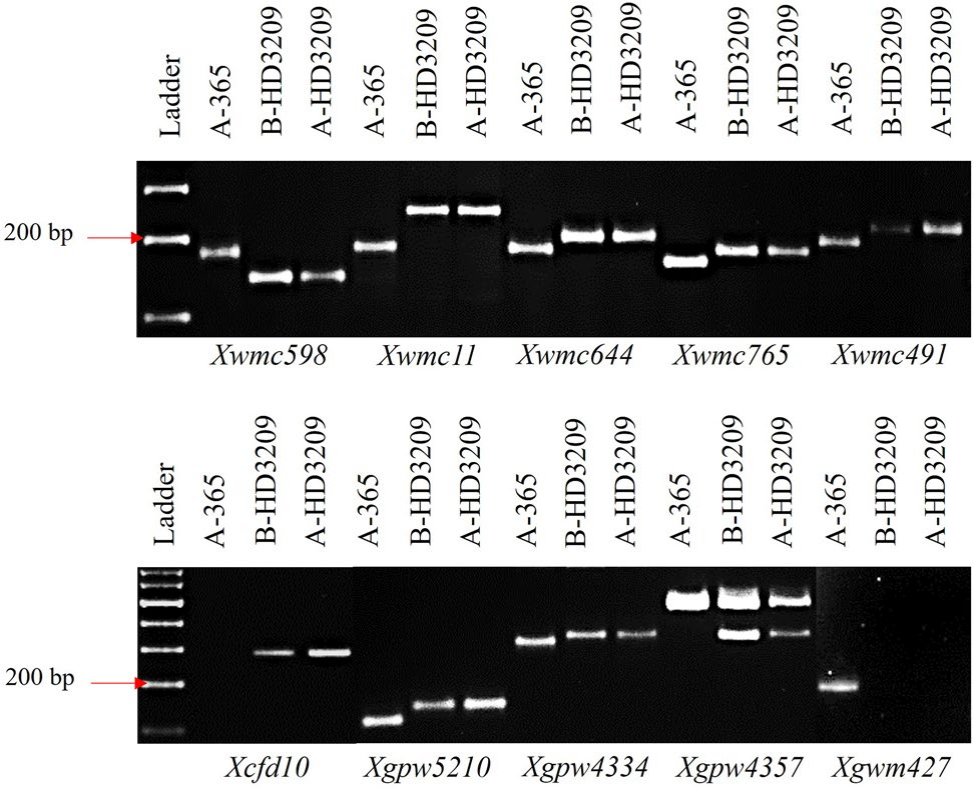
Amplification profile of 10 representative background polymorphic markers [from left to right: ladder (100 bp); P_1_, A-365; P_2_, B-HD3209, and derived CMS line A-HD3209].

**Figure 6:**
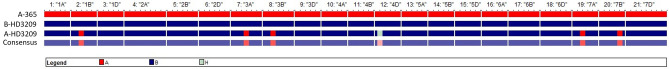
Graphical genotypes (GGT) describing recurrent parent genome recovery across the 21 chromosomes of derived CMS line A-HD3209.

All the backcross progenies of resistant recurrent parent with the dominant gene are supposed to be resistant as they may be homozygous or heterozygous resistant. Hence, it was essential to identify plants with homozygous resistant gene; otherwise, they may segregate for resistance in hybrids after crossing with restorer lines lacking resistance genes. Among the five BC_3_F_1_ plants tested for foreground markers *Xwmc221* and *Xgdm67,* four plants showed homozygous condition (Fig. 4). Among the plants homozygous for rust resistance, the best plant was selected based on agro-morphological traits and the BC_4_F_1_ progenies from the same selected plant was further used. To identify polymorphic SSRs for background analysis, 947 SSR markers across the 21 chromosomes of bread wheat A, B, and D-genomes were screened for parental polymorphism between donor A-365 and recurrent parent HD3209. One hundred and thirty-eight markers displayed presence/absence and/or length polymorphism between A-365 and HD3209. The polymorphic markers representing each chromosome arm were further employed for background analysis of BC_4_F_1_ plants (Fig. 5, Supplementary Fig. S1, Supplementary Table S1). These plants were also cross-verified for their phenotypic resemblance with their recurrent parent, HD3209 to minimize the plant-difference of CMS line A-HD3209. Background analysis was carried out using molecular markers in plants carrying target *Lr19/Sr25* genes and sterile cytoplasm in BC_4_F_1_ generations for each polymorphic locus of recurrent parent, HD3209-specific allele. In the BC_4_F_1_ generation of A-365 × HD3209 plants, 96.01% of the genome of the recurrent parent was recovered based on background analysis using molecular markers (Fig. 6). Plants exhibiting higher background genome recovery (BGR) for recurrent parent were identified and analysed for agro-morphological similarity.

### Agro-morphological similarity of A-line with recurrent parent

The A-HD3209 plants were tested for agro-morphological traits based on DUS testing guidelines and found to have almost complete similarity with recurrent parent HD3209, which will be further used as maintainer line, B-HD3209. The similarity with morphological traits indicated that the A-lines has recovered most genomic region from recurrent parent and have cytoplasm from donor parent with complete pollen sterility (Supplementary Table S2).

## Discussion

In hybrid breeding, while it is pre-requisite to select heterotic combinations, it is also important to integrate disease resistance in parental lines to reduce yield losses due to different diseases. In this context, the present study aimed at transferring *T. timopheevii*-derived sterile cytoplasm from CMS line A-365 to a superior genetic background, HD3209, which also carries resistance to leaf and stem rusts. The wheat genetic stock HD3209 is a near isogenic line (NIL) of wheat variety HD2932, carrying leaf and stem rust resistance genes *Lr19/Sr25*^9^. Wheat variety HD2932 is a high yielding variety of the central and peninsular zones of India. The NIL of HD2932, HD3209 also performed very well in All India Coordinated Research Project (AICRP) trials^26^, based on which it was taken for conversion into a CMS/A-line. According to Singh et al.^27^, the 7D.7Ag translocation improved grain yield potential across the genotypes by 10–15%. Hence, it was widely used in conventional and marker-assisted selection to incorporate leaf rust resistance.

The F_1_ plant between A-365 and HD3209 was completely sterile and has been backcrossed with the recurrent parent. The spike of backcrossed F_1_ plants have complete seed setting, which indicates typical stigma receptivity and standard seed setting in sterile plants on receiving sufficient pollen. Complete sterility and high seed setting on the crossing with pollen parent confirm the role of *timopheevii* cytoplasm in providing stable sterility and no undesirable effect on stigma receptivity. *Timopheevii* cytoplasm has been considered in many hybrid breeding programs and was advantageous over other cytoplasmic sources^20^. The backcrossed seeds were planted in field condition with the recommended package and practice. The seedlings were inoculated with mixed leaf rust pathotypes; as expected, all the seedlings were resistant. This happened because all the BC_1_F_1_ seeds were either *RR* or *Rr*, as the recurrent parent was *RR*.

All the progenies were resistant in the backcross generations because of a homozygous recurrent parent carrying the dominant gene *Lr19* for leaf rust resistance. The homozygous plants in BC_3_F_1_ generation were identified with the help of co-dominant linked markers *Xwmc221* (190 bp) and *Xgdm67* (129 bp). These markers were helpful in backcrosses breeding programs, and the gene has been successfully incorporated in many varieties to improve disease resistance^9,28^. The selection of homozygous plants was essential; otherwise, heterozygous plants may segregate in susceptible progeny on crossing with restorer lines not carrying any resistance gene and hence can show a mixture of susceptible plants on commercial cultivation. The selection in backcross generations were made based on simple visual agro-morphological traits like plant height, leaf orientation, overall plant architecture and days to heading compared to recurrent parent HD3209. The five most similar plants were selected in BC_1_F_1_, BC_2_F_1_ and BC_3_F_1_. The five selected plants of BC_3_F_1_ were tested for the presence of a resistance gene in homozygous conditions, and among the plants homozygous, the best plant was selected. The BC_4_F_1_ progenies and parents were further tested for rust resistance under controlled glasshouse conditions. The sterile plants were completely resistant, similar to the RP/B-line. The background analysis has shown the recovery of 96.01% of the recurrent parent genome. Theoretically the average background recovery in BC_4_F_1_ generation is 96.87%, but it is not always possible to capture that particular plant without any selection tools^29^. Phenotypic selection combined with marker-assisted backcrossing has been used successfully to develop NILs of wheat varieties HD2967 and HD2932^7,8^. The present results indicate that rigorous phenotypic selection in backcross generations can help in identifying plants with high recovery of the recurrent parent genome. Furthermore, the agro-morphological traits of the converted A-line were similar to recurrent parent. Additionally, the A-line has complete sterility and high seed setting on pollination, with the B-line indicating its likely usefulness in hybrid development depending on its heterotic combination with diverse restorer lines.

## Conclusion

Since the green revolution, several attempts have been made to develop hybrids in wheat crops, mainly using a cytoplasmic male sterility (CMS) system. However, hybrids have yet to be developed, having higher yields with disease resistance. The present study has converted superior wheat genotype HD3209 carrying *Lr19/Sr25* into CMS line A-HD3209 by crossing donor CMS line A-365 and recurrent parent HD3209. The converted CMS line A-HD3209 carrying *Lr19/Sr25* has complete resistance to leaf rust disease and has agro-morphological traits similar to recurrent parent HD3209. Hence, CMS line A-HD3209 can be utilized as a potential seed parent and, thus, be the first step towards hybrid development with rust resistance in wheat.

## Material and methods

### Plant materials

HD3209, a rust-resistant line carrying *Lr19/Sr25,* has been used as the recurrent parent. The recurrent parent has been developed through the rapid transfer of rust resistance genes in a wheat variety HD2932^9^, which was released and recommended for the late sown condition of the central and peninsular zones of India. CMS line A-365 has been used as the donor parent for sterile cytoplasm.

### Crossing and plan of conversion

The CMS line A-365 was used as a female parent and crossed with HD3209 as a pollen parent. The five F_1_ plants were backcrossed with recurrent parent, HD3209. The 100 BC_1_F_1_ seeds were grown, and five phenotypically selected plants were backcrossed with recurrent parent. The selection in BC_1_F_1_ was performed based on resistance to leaf rust, the similarity of the plant with recurrent parent and complete pollen sterility. The five plants having the most significant phenotypic similarity with recurrent parent, complete pollen sterility and rust resistance were selected and further backcrossed. Twenty seeds from each of the five BC_1_F_1_ plants were bulked, and 100 BC_2_F_1_ plants were grown. Similar criteria were used in BC_2_F_1_, and the five best plants were selected. Again, 100 BC_3_F_1_ plants were grown from the bulk of five selected plants from the previous generation. The five selected BC_3_F_1_ plants having complete sterility were identified for resistance genes in homozygous conditions through foreground analysis, and further the phenotypically best plant was selected for utilization in generation advancement and development of A-line (Table 1, Fig. 1).

### Evaluation for rust resistance in segregating backcross generations

The parental lines and backcrossed progenies under different generations till BC_3_F_1_ were tested with a mixture of *Puccinia triticina* pathotypes (77-5, 77-9, 12-5 and 104-2) under field conditions. Pure inoculum (uredospores) of pathotypes obtained from the ICAR-Indian Institute of Wheat & Barley Research, Regional Station, Flowerdale, Shimla were initially multiplied on susceptible Agra Local cultivars in a glass house at the Division of Genetics, ICAR-IARI, New Delhi. Under the field condition, backcrossed plants and the infector were evaluated for leaf rust at the adult plant stage. A mixture of susceptible cultivars, such as Agra Local, Kharchia Local and A9-30-1, were used as infector to spread the disease uniformly under field conditions. Approximately 55 days old plants were sprayed with a suspension of mixed inoculums prepared in water with a droplet of Tween-20. After 10–15 days of inoculation, individual plants of backcrossed segregating plants grown with infector rows were scored following a modified Cobb scale^30^.

### Molecular characterization for foreground and background analysis

To confirm the presence of the *Lr19/Sr25* genes in homozygous condition (BC_3_F_1_) and background recovery (BC_4_F_1_), the DNA was extracted from the selected plant using the CTAB method and the quality and quantity of DNA was confirmed on 0.8% agarose gel and Nanodrop spectrophotometer. The PCR reaction for SSRs was performed in a reaction volume of 10 μl, which included 4 μl of 2 × GoTaq PCR Master Mix (Promega, #M7122), 1 μl of each primer (5 pmol/μl), 2 μl of nuclease-free water, and 2 μl of 25 ng/μl gDNA (50 ng) in 96-well PCR plates with a thermal seal in an Eppendorf thermal cycler. A thermal profile of 4 min at 94 °C (initial denaturation), followed by 35 cycles of the 30 s at 94 °C (denaturation), 30 s at 50–60 °C (varies according to primer annealing temperature), 30 s at 72 °C (primer extension), and final extension at 72 °C for 10 min was used in a PCR machine for SSR marker amplification. On a 3.5% agarose gel, the amplified products were resolved and observed using a UV transilluminator Gel Documentation System (G: Box, Syngene). Recurrent parent genome (RPG) recovery was determined as the number of homozygous loci matching the recurrent parent plus half the number of heterozygous loci, divided by the total number of polymorphic SSR markers loci and multiplying the resultant by 100^23^.

Polymorphic SSR markers, i.e., *Xgdm67* and *Xwmc221,* closely linked to the *Lr19/Sr25* genes on chromosome 7DL of wheat, were used for foreground analysis in BC_3_F_1_ to confirm the presence of the genes in homozygous condition^9,28^. The background analysis was carried out on the bulked sample of 10 plants of BC_4_F_1_ from the selected best BC_3_F_1_ plant found to be homozygous for the resistance gene. The parental polymorphism between A-365 and HD3209 was firstly carried out for 947 wheat SSR markers showing genome-wide coverage of < 5 cM across the wheat genomes. Subsequently, the 138 polymorphic SSRs were employed for background analysis and recovery of recurrent parent genome was visualized using Graphical GenoTypes (GGT) Version 2.0 software^31^. Information regarding primer sequences and chromosomal location was obtained from GrainGenes (https://wheat.pw.usda.gov/GG3/), a database for Triticeae and Avena^32^.

### Pollen staining for analysis of male sterility

The CMS lines or A-lines used in developing hybrids should have 100% sterile pollen. The new CMS lines developed in the background of wheat genotype HD3209 needs to be evaluated for their sterility before using them in hybrid development. Considering the significance of pollen in defining CMS in plants, cytogenetic studies were performed to confirm the sterility of developed CMS lines against the parental genotype^33^. At the moment of anthesis, anthers from three florets were randomly selected, i.e., lower, middle, and upper sections of the primary spike of the male sterile and corresponding fertile line of recurrent parent. Primary spikes were sampled into 70% ethanol. Anthers were stained with 2% iodine-potassium iodide (I_2_-KI) solution for 2 min, followed by gently meshed with a pestle to release the pollen grains^34^, and visualized under the light microscope (Nikon Y-TV55, Japan). Digital photographs were captured with a Nikon Eclipse H600L camera (Nikon, Japan) fitted with the microscope. At least three independent biological replicates per sample were used for each experiment. Pollen grains that were totally spherical and intensely pigmented were considered fertile, but pollen grains that were unstained or stained but withered were classified as sterile^35^.

### Evaluation of BC_4_F_1_ plants for agro-morphological traits

To record the agro-morphological traits of DUS, the final converted A-line in BC_4_F_1_ generation was grown alongside recurrent parent HD3209, which will function as the B-line for maintaining the A-line. The A and B-lines were raised at a plant-to-plant distance of 5 cm and a row-to-row distance of 22.5 cm using the specified package and practice. The DUS characteristics were recorded at an appropriate stage following the manual of DUS testing (https://plantauthority.gov.in/crop-dus-guidelines).

### Phenotyping for leaf rust resistance and single-race testing of A-HD3209

For the single race testing (SRT), the parental lines and BC_4_F_1_ progenies from the selected BC_3_F_1_ plant were tested at the seedling stage against the most prevalent Indian pathotypes 77-5, 77-9, 104-2 and 12-5 under the glasshouse condition*.* Ten-day-old seedlings were evaluated for leaf rust resistance by inoculating pure inoculum of *P. triticina*. Inoculated seedlings were first incubated for 48 h in a humid chamber. After incubation, seedlings were transferred to the glass house at a temperature of 16 °C under ambient light and humid conditions^2^. For infection types (ITs), individual seedlings were scored following a 0–4 scale after 10–12 days of inoculation^36^.

### Formal identification of the plant material

All plant materials used in the current study are the popular wheat varieties developed by the ICAR-Indian Agricultural Research Institute, New Delhi.

### Statements of guidelines and regulation

All methods have been used/performed in accordance with the well-established guidelines and regulations.

### Supplementary Information

Supplementary Information.

## Data Availability

The current study has not generated any molecular datasets. The analysed phenotypic data have been included in the supplementary section.
