# Supplementary material for: Conversion of superior bread wheat genotype HD3209 carrying Lr19/Sr25 into CMS line for development of rust-resistant wheat hybrids
Source: Sci Rep. 2024 Jun 19;14:14112. doi: 10.1038/s41598-024-65109-x (PMC11187221; doi:10.1038/s41598-024-65109-x)
Supplement: Supplementary file 1 — Supplementary Information. [file 41598_2024_65109_MOESM1_ESM.docx]

**Supplementary Table S1**. Details of 138 polymorphic SSR markers distributed across the 21 chromosomes of A, B, and D subgenomes of derived CMS line A-HD3209

| 1A | *Xgwm136* | 2D | *Xcfd10* | 4B | *Xgpw4120* | 6A | *Xgwm459* | 7D | *Xwmc221* |
| --- | --- | --- | --- | --- | --- | --- | --- | --- | --- |
|  | *Xgpw2142* |  | *Xgpw361* |  | *Xgpw7390* |  | *Xgpw2295* |  | *Xgdm67* |
|  | *Xwmc744* |  | *Xgpw5090* |  | *Xgpw4175* |  | *Xgwm427* |  | *Xwmc634* |
|  | *Xwmc11* |  | *Xgpw5215* |  | *Xgwm495* |  | *Xwmc553* |  | *Xcfd69* |
|  | *Xgpw4334* |  | *Xgpw2321* |  | *Xgwm6* |  | *Xgpw4142* |  | *Xgpw3045* |
|  | *Xcfa2147* |  | *Xgpw5163* |  | *Xgpw4150* |  | *Xgpw5125* |  | *Xgpw5054* |
| 1B | *Xgwm273* |  | *Xcfd44* | 4D | *Xgpw5133* | 6B | *Xgpw4395* |  | *Xgpw5181* |
|  | *Xgwm18* | 3A | *Xgwm2* |  | *Xcfd23* |  | *Xcfd1* |  | *Xgpw5279* |
|  | *Xwmc597* |  | *Xgwm32* |  | *Xcfd81* |  | *Xwmc737* |  | *Xgpw4129* |
|  | *Xgwm498* |  | *Xgwm5* |  | *Xwmc720* |  | *Xwmc726* |  | |
|  | *Xgwm582* |  | *Xwmc594* |  | *Xgpw5314* |  | *Xgpw2060* |  |  |
|  | *Xgwm268* |  | *Xgpw7219* |  | *Xcfd54* |  | *Xgpw4138* |  |  |
| 1D | *Xgdm126* |  | *Xwmc264* | 5A | *Xgpw3003* |  | *Xgpw5076* |  |  |
|  | *Xcfd65* |  | *Xcfa2183* |  | *Xgwm415* | 6D | *Xgpw4357* |  |  |
|  | *Xgpw5260* | 3B | *Xcfd143* |  | *Xcfa2185* |  | *Xcfd76* |  |  |
|  | *Xwmc216* |  | *Xwmc754* |  | *Xgpw2181* |  | *Xwmc753* |  |  |
|  | *Xgwm642* |  | *Xwmc544* |  | *Xwmc524* |  | *Xgpw1101* |  |  |
|  | *Xgpw5162* |  | *Xwmc679* |  | *Xcfd47* |  | *Xgpw4309* |  |  |
| 2A | *Xwmc407* |  | *Xgpw3156* | 5B | *Xgpw7244* |  | *Xgpw362* |  |  |
|  | *Xwmc598* |  | *Xwmc762* |  | *Xgwm293* |  | *Xgpw5210* |  |  |
|  | *Xwmc644* |  | *Xwmc274* |  | *Xgwm66* | 7A | *Xwmc497* |  |  |
|  | *Xwmc794* | 3D | *Xwmc674* |  | *Xgwm499* |  | *Xwmc83* |  |  |
|  | *Xgpw3251* |  | *Xwmc533* |  | *Xgwm408* |  | *Xwmc65* |  |  |
|  | *Xwmc702* |  | *Xgpw5213* |  | *Xgwm271* |  | *Xcfa2123* |  |  |
|  | *Xwmc632* |  | *Xwmc552* | 5D | *Xgpw5189* |  | *Xcfa2293* |  |  |
|  | *Xgpw2253* |  | *Xcfd152* |  | *Xgpw4020* |  | *Xcfa2240* |  |  |
|  | *Xcfd50* |  | *Xcfd70* |  | *Xcfd37* | 7B | *Xgpw5134* |  |  |
| 2B | *Xcfa2278* | 4A | *Xgpw2140* |  | *Xwmc765* |  | *Xcfd74* |  |  |
|  | *Xgpw4390* |  | *Xcfa2026* |  | *Xcfd29* |  | *Xcfd22* |  |  |
|  | *Xgpw4396* |  | *Xcfd16* |  | *Xcfd25* |  | *Xgwm274* |  |  |
|  | *Xcfa2201* |  | *Xwmc491* |  | |  | *Xwmc723* |  |  |
|  | *Xgwm410* |  | *Xgpw2331* |  |  |  | *Xgpw8090* |  |  |
|  | *Xgpw4043* |  | *Xwmc722* |  |  |  | |  |  |
|  | *Xgpw4474* |  | |  |  |  |  |  |  |

Cfa, Cleromont-Ferrand A-genome; cfd, Cleromont-Ferrand D-genome; gdm, Gatersleben D-genome microsatellites; gwm, Gatersleben wheat microsatellites; gpw, Wheat genoplante; and wmc, Wheat microsatellite consortium

**Supplementary Table S2**. Details of agromorphological characteristics of A-HD3209, B-HD3209, and A-365 lines studied for DUS traits under field conditions.

| **S. No.** | **DUS Traits** | **A-HD3209** | **B-HD3209** | **A-365** |
| --- | --- | --- | --- | --- |
|  | Coleoptile: Anthocyanin colouration | 1 | 1 | 1 |
|  | Plant: Growth habit | 3 | 3 | 3 |
|  | Foliage: Colour | 5 | 5 | 1 |
|  | Flag leaf: Anthocyanin colouration of auricles | 1 | 1 | 5 |
|  | Flag leaf: Hairs on auricle | 5 | 5 | 3 |
|  | Plant: Flag leaf attitude | 5 | 5 | 3 |
|  | Ear: Time of emergence (first spikelet visible on 50% of ears) | 5(94 days) | 5(93 days) | 5 |
|  | Flag leaf Waxiness of sheath | 3 | 3 | 3 |
|  | Ear: Waxiness | 5 | 5 | 5 |
|  | Culm: Waxiness of neck (Peduncle) | 5 | 5 | 7 |
|  | Flag leaf: Length | 9 | 9 | 1 |
|  | Flag leaf: Width | 5 | 5 | 1 |
|  | Plant: Length excluding awns/scurs) | 3 | 3 | 3 |
|  | Ear: Shape in profile | 2 | 2 | 5 |
|  | Ear: Density | 3 | 3 | 5 |
|  | Ear: Length (excluding awns and scurs) | 3 | 3 | 5 |
|  | Awns or scurs: Presence | 3 | 3 | 3 |
|  | Scurs | 1 | 1 | 1 |
|  | Awns: Length | 3 | 3 | 3 |
|  | Awn: Colour | 2 | 2 | 3 |
|  | Awn: Attitude | 1 | 1 | 2 |
|  | Outer glume: Pubescence | 5 | 5 | 5 |
|  | Ear: Colour | 1 | 1 | 2 |
|  | Lower glume: Shoulder shape (spikelet in mid-third of ear) | 5 | 5 | 5 |
|  | Lower glume: Beak length | 5 | 5 | 3 |
|  | Lower glume: Beak shape (spikelet in mid-third of ear) | 1 | 1 | 1 |
|  | Peduncle: Length | 5 | 5 | 5 |
|  | Spike attitude (at the time of flowering) | 1 | 1 | 1 |
|  | Grain: Colouration with phenol | 7 | 7 | 9 |
|  | Grain: Colour | 2 | 2 | 2 |
|  | Grain: Shape | 3 | 3 | 3 |
|  | Grain: Germ width | 5 | 5 | 5 |
|  | Brush hair: Length | 5 | 5 | 5 |
|  | Seed: Size (weight of 1000 grains) | 3(40.96 g) | 3(41.56 g) | 3 |
|  | Season: Type | 1 | 1 | 1 |
|  | Grain: Hardness | 5 | 5 | 5 |
| Additional | No. of Spikelet/Spike | (18.59) | (19) | - |

DUS characteristics were recorded at an appropriate stage following the manual of DUS testing (<https://plantauthority.gov.in/crop-dus-guidelines>)

**Supplementary Fig. S1**. Chromosomal localization of 138 polymorphic SSR markers distributed across the 21 chromosomes of A, B, and D genome of derived CMS line A-HD3209


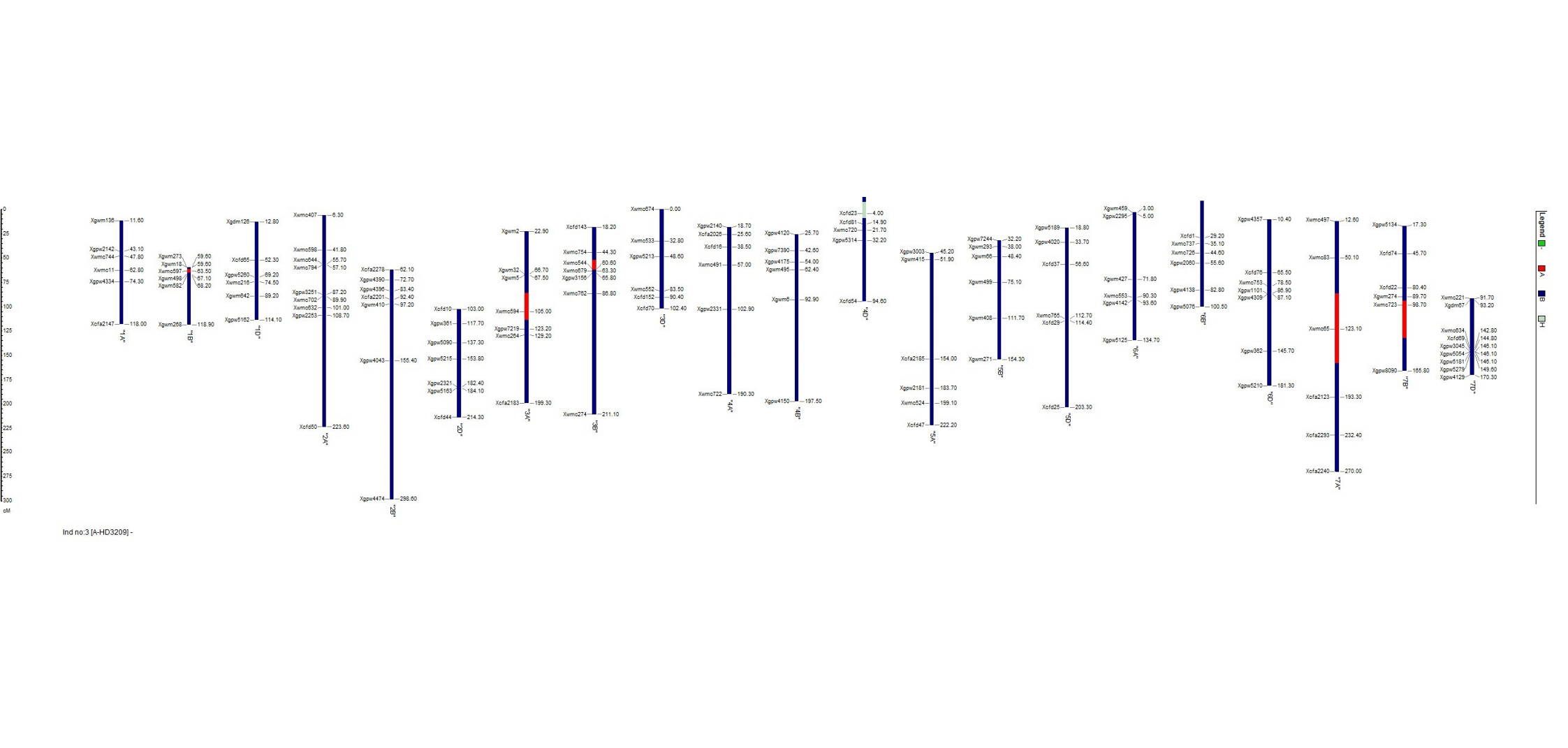


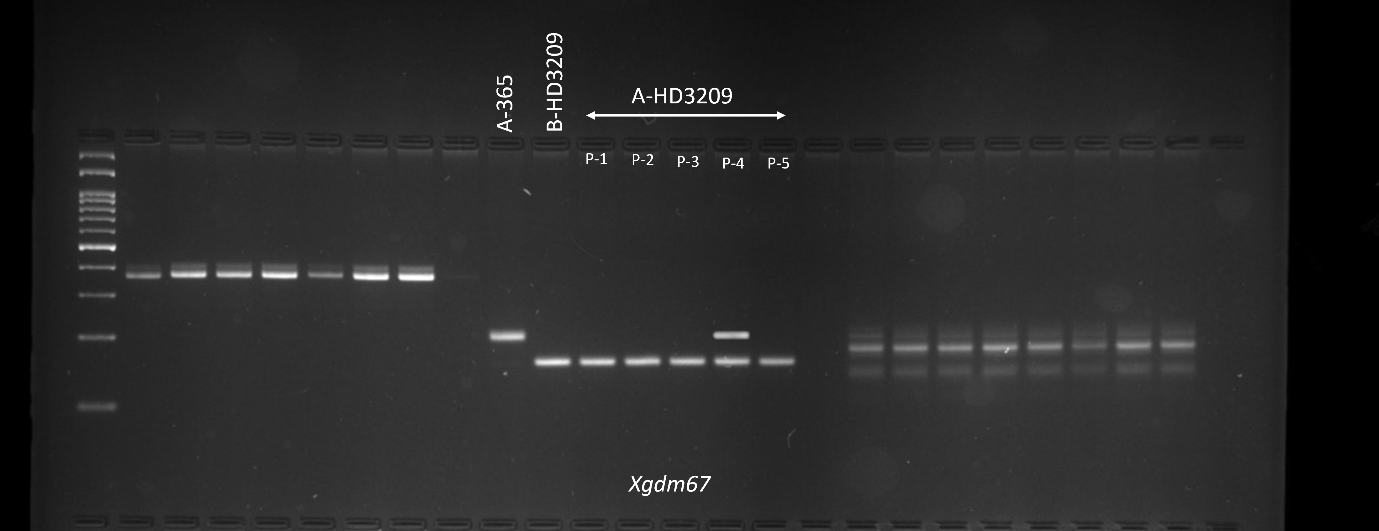


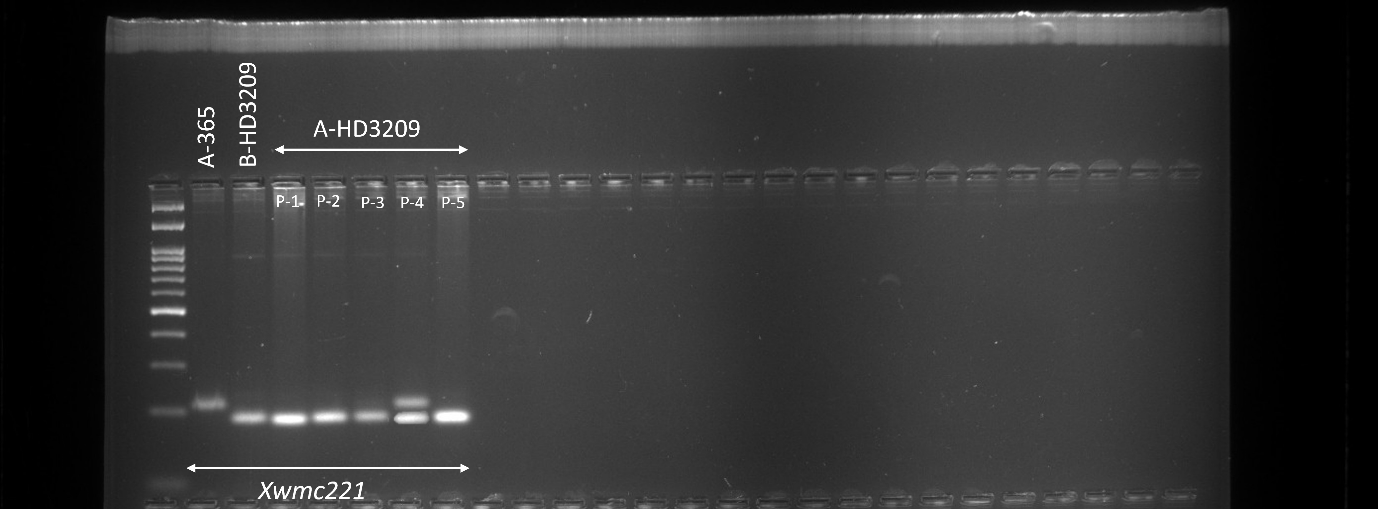


Uncropped images for Fig. 4


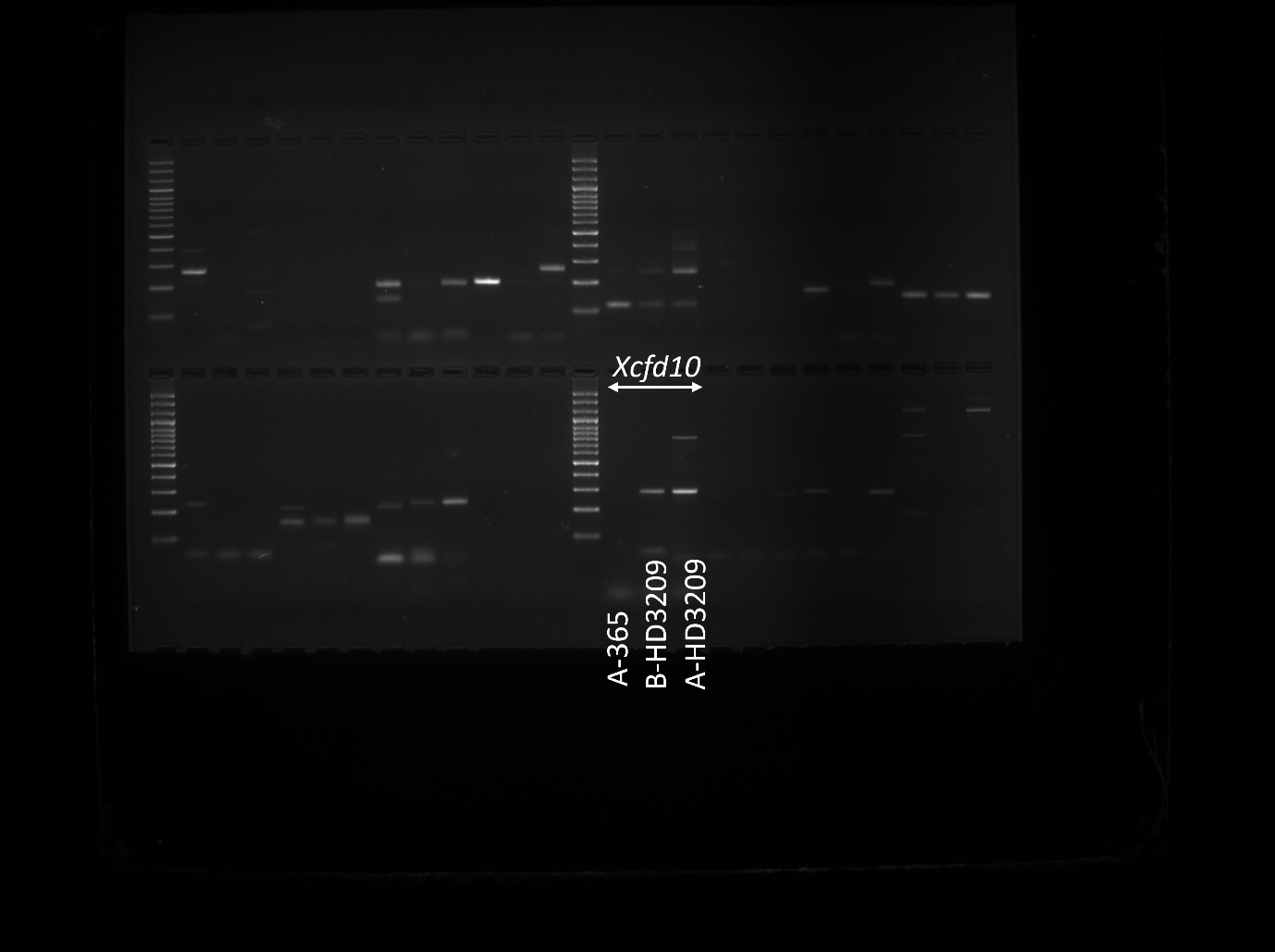


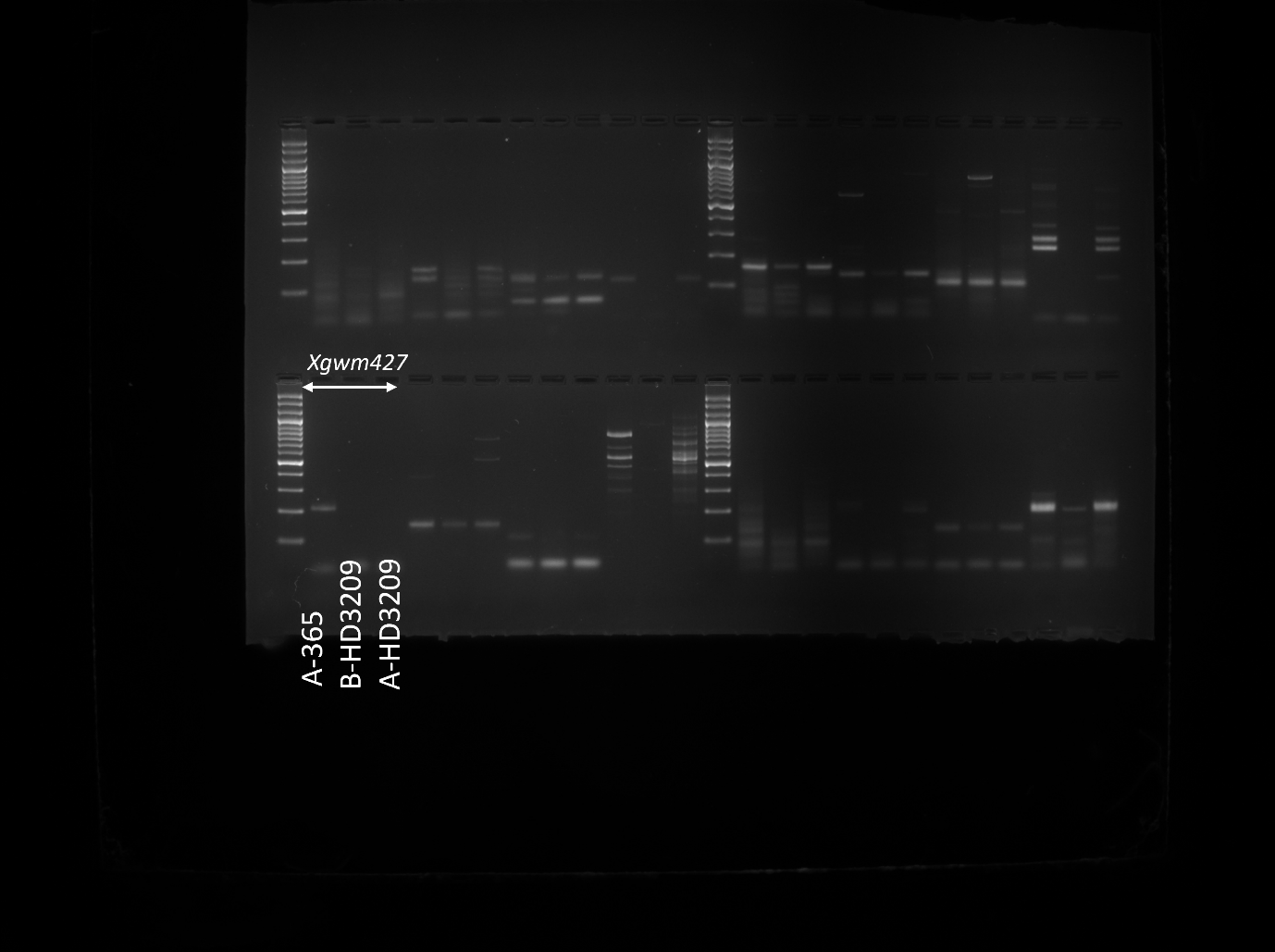


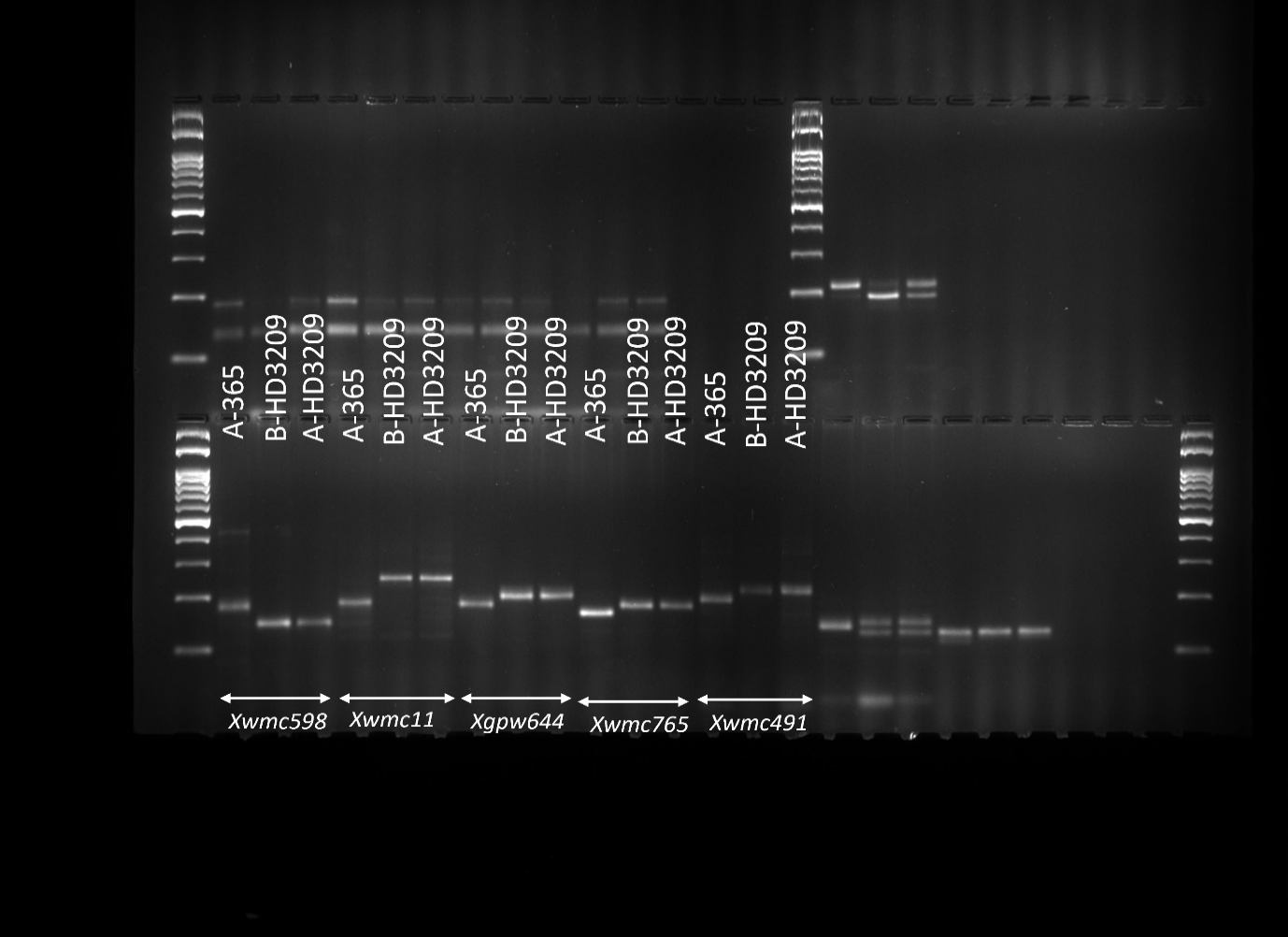


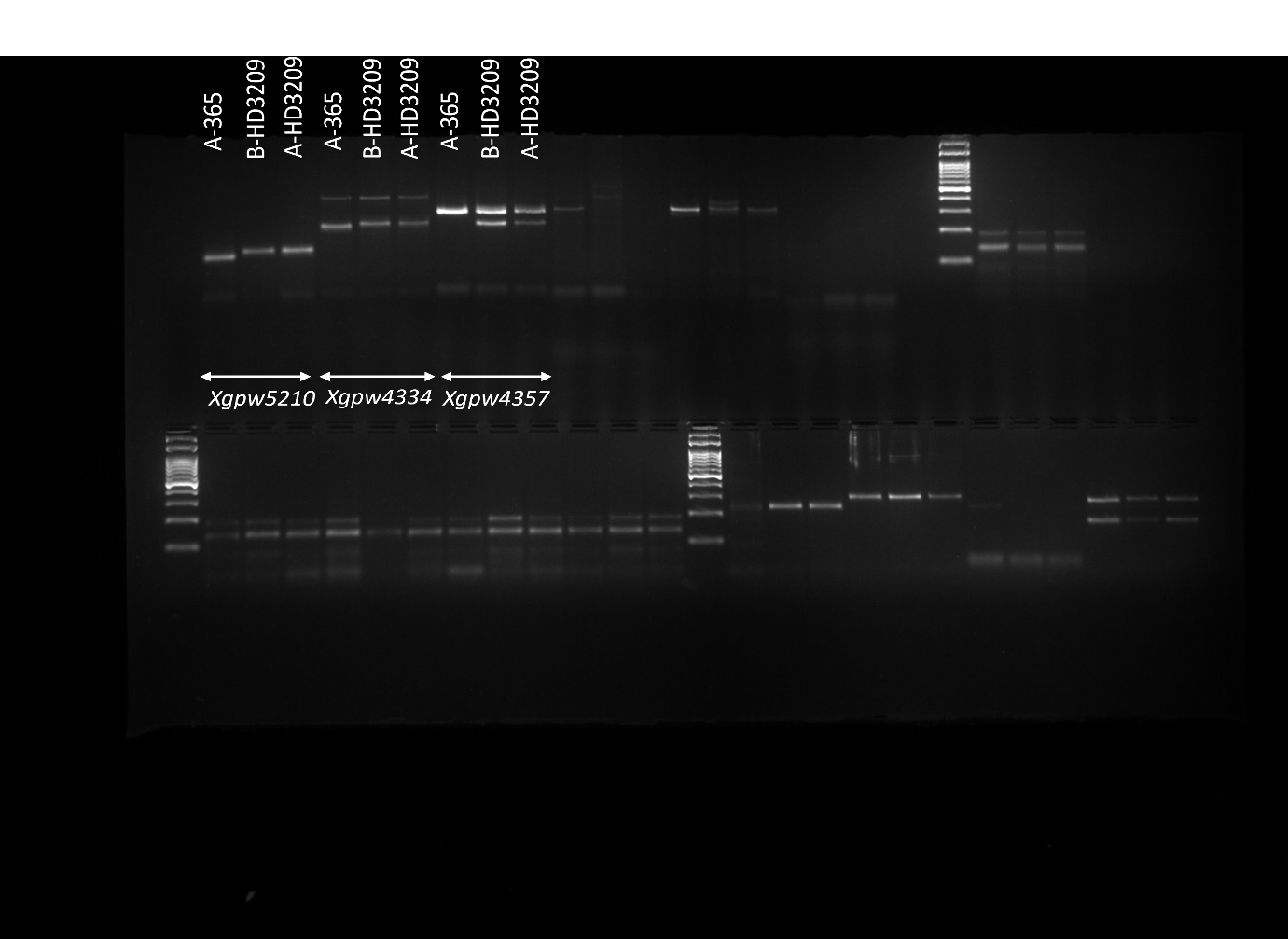


Uncropped images for Fig. 5
